# *Macrophomina phaseolina* expresses sequential waves of effector, secondary metabolite, and CAZyme genes during strawberry infection, suggesting a hemibiotrophic lifestyle

**DOI:** 10.3389/fmicb.2026.1856252

**Published:** 2026-08-31

**Authors:** Christine Jade Dilla-Ermita, Kayla K. Pennerman, Marta Bjornson, Olivia Gutierrez, Zoey Jimenez, Gerardo Ramos, Polly Goldman, Lacey Prescott, Javier Lopez-Hernandez, Alicia Sillers, Mitchell J. Feldmann, Peter M. Henry

**Affiliations:** 1 Department of Plant Sciences, University of California Davis, Davis, CA, United States; 2 Crop Improvement and Protection Research Unit, USDA-ARS, Salinas, CA, United States; 3 Biology Program, Stockton University, Galloway, NJ, United States; 4 Department of Plant Pathology, University of California Davis, Davis, CA, United States

**Keywords:** *Fragaria x ananassa*, *Macrophomina phaseolina*, *Macrophomina* root rot, pathogenicity, temperature-dependent, transcriptional changes

## Abstract

*Macrophomina phaseolina* causes crown and root rot disease in strawberry (*Fragaria* *ananassa*) and has become more damaging with increasing temperatures in strawberry-growing areas worldwide. Little is known about the molecular events during *M. phaseolina* infection in strawberry, such as transcriptional changes at elevated temperatures and different stages of infection. To fill this knowledge gap, we analyzed transcriptional changes during *M. phaseolina* infection in strawberry plants grown in controlled environments with high (30°C) and low (23°C) daytime temperature regimes. At high temperatures, plants had more severe crown/root necrosis, wilting, and accelerated disease progress compared to plants incubated at low temperature. Based on the composite analysis of the temperature treatments, transcriptional changes in *M. phaseolina* and strawberry were greatly influenced by disease stage, with each stage having distinct expression patterns of specific gene classes. In *M. phaseolina*, effector-encoding genes were highly enriched and expressed at the early disease stage, whereas during necrotrophy at mid-infection, secondary metabolite-encoding genes were highly upregulated. Carbohydrate active enzyme (CAZyme) expression was enriched at late infection. These patterns of gene enrichment and expression suggest *M. phaseolina* has a hemibiotrophic lifestyle during strawberry infection. Under high temperature, *M. phaseolina* pathogenicity-associated genes were upregulated while immunity-related genes in strawberry were downregulated, suggesting that increased disease severity under this treatment is at least partially attributed to susceptibility-related plant responses. Our study is the first to characterize the *M. phaseolina*-strawberry interaction using transcriptomics and to investigate mechanisms by which high temperature affects *M. phaseolina* pathogenesis.

## 1 Introduction

Plant pathogens are categorized into three distinct lifestyles based on the viability of host cells from which they acquire nutrients to survive. The first group are biotrophs, which acquire nutrients from living host cells, often through specialized structures such as haustoria. Biotrophs typically secrete few cell wall-degrading enzymes (CWDEs) and generally do not produce toxins (Kabbage et al., 2015). On the other extreme, necrotrophs acquire nutrients by killing host cells through the secretion of toxins (Walton, 1996; Friesen et al., 2008), cell death-inducing effectors (Shao et al., 2021), induction of host reactive oxygen species (ROS) production (Govrin and Levine, 2000) and secretion of large repertoires of CWDEs (Horbach et al., 2011). Hemibiotrophs utilize aspects of both lifestyles, where the pathogen has an initial biotrophic phase in which infection occurs without killing host cells and later switches to necrotrophy (Kabbage et al., 2015).

A growing number of studies on fungal pathogens that were previously considered to be necrotrophs have revealed that a biotrophic phase occurs during the early infection stage (Horbach et al., 2011; Rajarammohan, 2021). For example, compelling molecular and cytological evidence in *Sclerotinia sclerotiorum* (Kabbage et al., 2013, 2015; Chittem et al., 2020) supports a two-phase infection model (Liang and Rollins, 2018) with a brief biotrophic phase during early infection when the plant’s immune response is subverted, followed by a typical necrotrophic phase. *Sclerotinia sclerotiorum* produces thickened hyphae in the apoplastic space, without causing death during early infection (Kabbage et al., 2015), while producing oxalic acid (OA) that suppresses oxidative burst-associated cell death (Williams et al., 2011). Along with OA, biotrophy-associated effectors such as chorismate mutase (SsCm1), proteins with LysM (lysin motif), other chitin-binding proteins, and ROS-scavenging genes are highly expressed at the early infection phase (Chittem et al., 2020). After establishing an infection, the fungus switches to necrotrophy through upregulation of necrosis-inducing effectors including cerato-platanin, small cysteine rich secreted proteins, and necrosis and ethylene-inducing peptides at late infection (Chittem et al., 2020). Simultaneously, OA accumulates, leading to activation of CWDE expression and generation of plant ROS that can cause plant cell death (Williams et al., 2011; Kabbage et al., 2013).

A similar infection strategy was observed in the hemibiotrophic fungal pathogens *Colletotrichum higginsianum*, *C. graminicola*, and *C. orbiculare* (O’Connell et al., 2012; Gan et al., 2013). These pathogens have an upregulation of genes for effectors and secondary metabolite enzymes during the early stage of infection (biotrophy), while CWDEs such as hydrolases are upregulated at a later stage, when the pathogens have switched to necrotrophy (O’Connell et al., 2012; Gan et al., 2013). Expression profiling and cytological analysis revealed that *C. higginsianum* expresses and secretes biotrophy-associated effectors that suppress plant cell death during early infection processes, then upregulates cell-death inducing effectors such as ChNLP1 and ChToxB during the switch to necrotrophy (Kleemann et al., 2012). These studies demonstrate that specific infection stages involve expression of different suites of genes and there is a tightly coordinated transition between different infection stages.

For years, *Macrophomina phaseolina* has been considered a necrotrophic fungus (Islam et al., 2012; Sarkar et al., 2014; Bandara et al., 2018) that infects a wide range of plant hosts (Pennerman et al., 2024). It is globally prevalent and destructive (Songa and Hillocks, 1996; Wrather et al., 2001; Sarr et al., 2014), especially in regions with dry and warm climates (Kaur et al., 2012; Cohen et al., 2016; Pandey and Basandrai, 2020). Recently, *M. phaseolina* has become an increasingly important problem in strawberry (*Fragaria x ananassa*) production worldwide, causing a crown and root rot disease that kills plants and significantly decreases yield (Baudry and Morzieres, 1993; Zveibil and Freeman, 2005; Golzar et al., 2007; Avilés et al., 2008; Koike, 2008; Baino et al., 2011; Sánchez et al., 2013). Highly resistant strawberry genotypes to *M. phaseolina* are rare and only account for 1% of the strawberry wild and cultivated germplasm (Knapp et al., 2024). There can be an extended interval between asymptomatic infection in the roots and the onset of symptoms in strawberry (Baggio et al., 2019; Pedroncelli et al., 2025). This suggests that the plant is first asymptomatically colonized by *M. phaseolina*, and disease expression occurs later, when the environmental conditions become favorable for pathogenesis. When there are favorable conditions for pathogenesis and poor disease management practices, the disease can cause greater than 80% plant mortality (Peres and Mertely, 2007; Baggio et al., 2019).

Elevated temperatures increase the severity of diseases caused by *M. phaseolina* in soybean (Teja et al., 2020), melon (de Sousa Linhares et al., 2020; Cohen et al., 2022) and chickpea (Sharath Chandran et al., 2021). Likewise in strawberry, elevated temperatures increase disease severity (Fang et al., 2011; Wang Y. C. et al., 2024) and high soil temperature (30°C) increase plant mortality due to *Macrophomina* crown and root rot (Zveibil et al., 2012). This high temperature-induced disease severity and plant mortality can be attributed to the adaptability of *M. phaseolina* to warm climates; it has an optimum temperature ranging from 30°C to 35°C for fungal growth (Manici et al., 1995) and microsclerotia production (Akhtar et al., 2011). Furthermore, plant susceptibility to *M. phaseolina* increased under high temperature was reported in melons (de Sousa Linhares et al., 2020; Cohen et al., 2022), chickpea (Sharath Chandran et al., 2021) and strawberry (Wang Y. C. et al., 2024). Under high temperature, significant downregulation of phenylalanine ammonia-lyase (PAL) and peroxidase genes compared to low (25°C) temperature was observed in a resistant chickpea genotype, suggesting that high temperature impaired phytoalexin biosynthesis and reactive oxygen species (ROS) metabolism, leading to plant susceptibility (Sharath Chandran et al., 2021).

A study on sesame challenged the notion that *M. phaseolina* is a necrotrophic pathogen by showing that early infection events have the hallmarks of biotrophy with a later switch to necrotrophic gene expression (Chowdhury et al., 2017). During the early stage of infection, *M. phaseolina* produced intercellular hyphae and intracellular vesicles, and had upregulation of a gene encoding for a biotrophy-associated secreted protein (*BAS3*). Once *M. phaseolina* switched to necrotrophy, necrotrophy-inducing protein (*NIP*) was upregulated and *BAS3* was downregulated, concurrent with the growth of secondary necrotrophic hyphae and loss of cell viability (Chowdhury et al., 2017). These results countered the common belief that *M. phaseolina* is a necrotrophic fungus but focused on a limited number of genes in both the host and pathogen.

Despite extensive reports of *M. phaseolina* in strawberries, molecular interactions between *M. phaseolina* and strawberry during infection have not been characterized. Analysis of the *M. phaseolina* genome from a jute isolate (Islam et al., 2012) revealed an abundance of CWDE-encoding genes and secondary metabolite genes associated with a necrotrophic lifestyle. Burkhardt et al. (2019) compared the genomes of *M. phaseolina* isolates from strawberry, alfalfa, and jute, and found thousands of orthologous clusters common among the three genomes that were enriched with Gene Ontology (GO) terms associated with oxidoreductase activity and terpenoid biosynthetic processes. These genomic investigations provide a foundation for characterizing the molecular events during M. phaseolina-strawberry interaction.

Here, we performed RNAseq experiments and characterized the transcriptional changes in *M. phaseolina* and strawberry throughout the course of infection under different temperature treatments. We aimed to characterize the molecular events during *M. phaseolina*-strawberry interaction and to determine how high temperatures influence disease progress and transcriptional changes in both the host and the pathogen. To achieve this, we used susceptible strawberry genotypes to be able to observe the full range of molecular events of *Macrophomina* pathogenesis and understand how pathogen virulence impacts plant transcriptome. Disease progress and transcriptional expression were evaluated under high and low temperature regimes to understand the effects of temperature in fungal virulence and plant susceptibility. We determined that high temperatures accelerate disease progress of *M. phaseolina*. We found that pathogenicity-associated gene classes (CAZymes, effectors and secondary metabolites) were enriched and expressed in waves structured by disease stage, from the early infection stage to the saprotrophic stage. On the other hand, we did not observe distinct disease stage-specific enrichment of phytohormone, CAZyme and pathogenesis-related (PR) genes in strawberry. The disease stage-specific expression of specific genes in *M. phaseolina* supports the characterization of this fungus as a hemibiotroph. Further examination of the effects of temperature in the transcriptome of *M. phaseolina* and strawberry revealed high temperature-dependent upregulation of pathogenicity-associated genes in *M. phaseolina*. Simultaneously, high temperatures led to downregulation of immunity-related genes in the two susceptible strawberry varieties (Monterey and San Andreas) tested in our study.

## 2 Materials and methods

### 2.1 Growth chamber experiments and pathogenicity assessments

Growth chambers were set to two temperature regimes with a 12-hour light cycle: 23°C day/18°C night temperature (low) and 30°C day/25°C night temperature (high). We did not control for relative humidity but the average ambient relative humidity ranges from 66 to 74% in Salinas, California where we conducted our experiments. The experiments were conducted with two temperature regimes, four timepoints (1, 5, 12, and 21 days post-inoculation) and two treatments (*M. phaseolina* F005 isolate and uninoculated control). Each treatment combination was replicated five times. The entire experiment, including RNA extraction and sequencing, was conducted twice.

We used *M. phaseolina* F005 isolate in this study as it is clonally related to the majority of strawberry-derived *M. phaseolina* strains (Pennerman et al., 2025). Inoculum preparation and inoculation of *M. phaseolina* was done following the protocol described by Pennerman et al. (2025). A single 5-mm diameter plug from a one-week-old culture of *M. phaseolina* F005 isolate on potato dextrose agar (with 50 μg/ml kanamycin, 100 μg/ml streptomycin, and 12.5 μg/ml chlortetracycline) was inoculated into a 500 ml flask containing 200 ml of 10% V8 broth. The five inoculated flasks were incubated in the dark at 25°C and shaken at 200 rpm for 3 days. The mycelial suspension was then blended at high speed for 7 s. To determine the inoculum concentration, 25 ml of the suspension was transferred to a 50 ml conical tube and was left to settle for 3–5 min to measure the percentage of mycelium. The mycelial suspension was diluted with sterile DI water to 8% mycelia.

Growth chamber experiments were conducted twice using two highly susceptible, market-relevant strawberry varieties (Knapp et al., 2024): San Andreas for Trial 1 and Monterey for Trial 2. Month-old strawberry daughter plants (*n* = 40) were inoculated with *M. phaseolina* by immersing roots in the 8% mycelial suspension for 10 min. For the non-inoculated controls, another set of 40 daughter plants were dipped in diluted sterile V8 broth (10% V8 broth diluted to the same ratio used to dilute the inoculated V8 broth) in each trial. Daughter plants were planted in 8.25 × 8.25 × 9 cm (L × W × H) pots containing twice autoclaved Sakrete Natural Play Sand (Sakrete, Sandy Springs, GA, USA). Excess inoculum or diluted V8 broth (approximately 25 ml) was added to corresponding pots.

At 1, 5, 12, and 21 days post-inoculation (dpi), plant symptoms were documented and root/fungal tissue was collected. Scoring of above-ground disease severity was done for all five replicates per treatment combination for each trial, and was based on a modified 1–5 scale described by Koike et al. (2016): 1 - no symptoms; 2 - mild stunting or only lower older leaves wilted, chlorotic, and/or necrotic; 3 - less than 50% of the leaves wilted, chlorotic, and/or necrotic with symptomatic younger leaves; 4 - more than 50% of the leaves wilted, chlorotic, and/or necrotic; 5 - complete plant collapse or death. Composite statistical tests of disease scores for the two trials were done using six or eight replicates per treatment combination. Statistical differences in disease scores among different treatment combinations were evaluated using the Kruskal-Wallis test (*p* < 0.05). To determine significant differences between high and low temperatures per timepoint, the Wilcoxon Rank Sum two-sided test in R was used.

**FIGURE 1:**
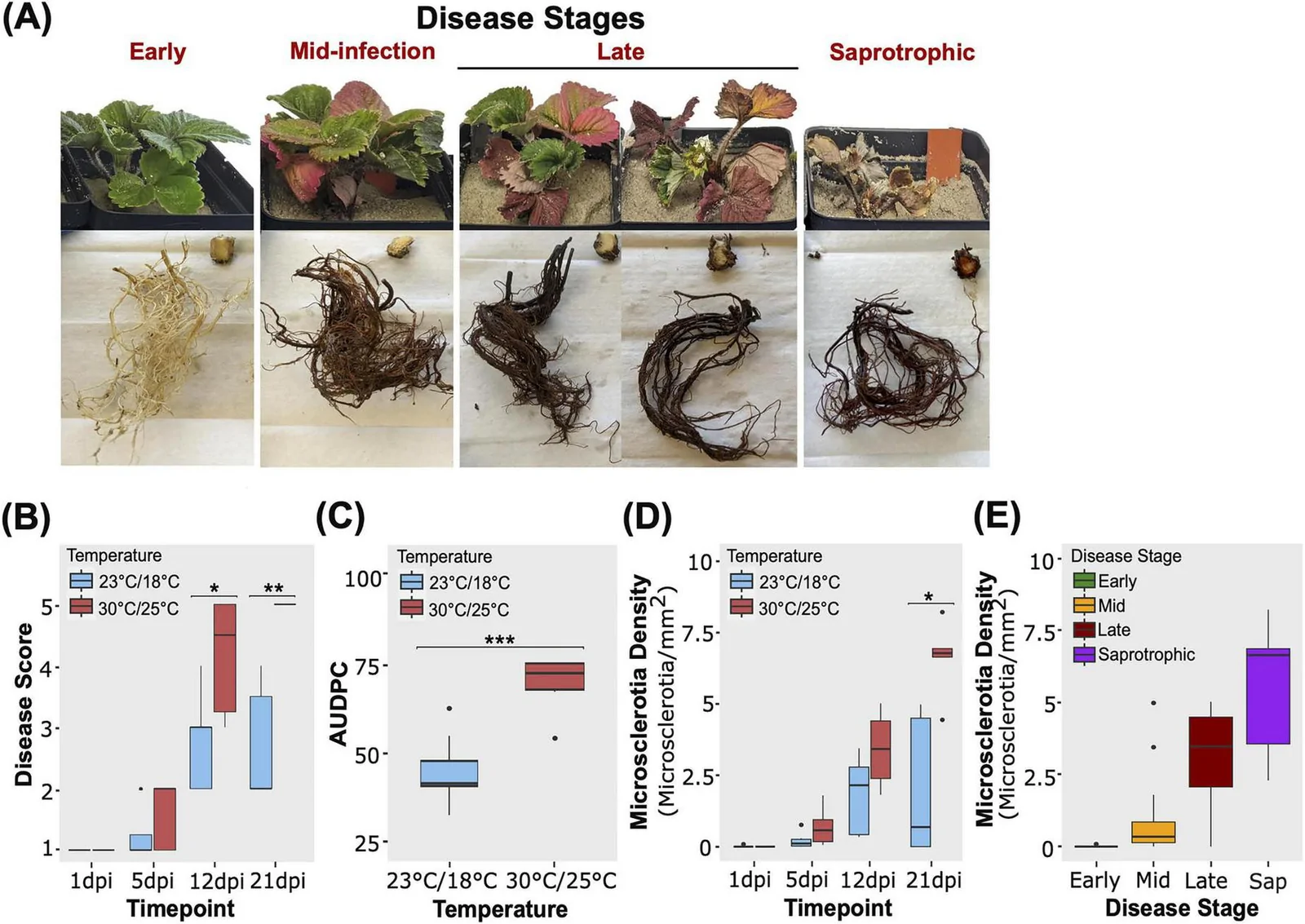
Disease stages and disease severity of *Macrophomina* infection in strawberry. **(A)** Plant samples classified by disease stage: early infection - no aboveground symptoms, and no crown or root necrosis; mid-infection- mild stunting, chlorosis or reddening of leaves, root necrosis and/or mild crown discoloration; late infection- dieback and/or wilting, crown discoloration and severe root necrosis; saprotrophic- dead plants and severely necrotic crowns and roots. Composite analysis of **(B)** disease severity scores **(C)** area under disease progress curve (AUDPC), and microsclerotia density (internal microsclerotia per mm^2^ of roots) of *Macrophomina phaseolina* **(D,E)** determined by microscopy using Olympus BX53 microscope. The number of internal microsclerotia was manually counted and sided root area (mm^2^) was measured using Olympus cellSens Standard 4.1 software at different temperatures **(D)** and disease stages **(E)**, evaluated until 21 dpi for two trials. Each treatment combination had six to eight replicates for composite analysis of the two trials. Significant difference of high temperature over low temperature per timepoint in B-D based on Wilcoxon Rank Sum two-test was annotated with asterisk (*, **, *** indicate *p* < 0.05, 0.01, 0.001, respectively).

To capture the overall disease progress in both above-ground and below-ground tissues, we also assigned plant samples to disease stages (Figure 1A) based on above-ground symptoms corresponding disease scores and the presence of root and crown necrosis (tissue browning as evidence of plant cell death): early infection - no aboveground symptoms, and no crown and root necrosis; mid-infection – mild stunting, chlorosis or reddening of leaves, root necrosis and/or mild crown discoloration; late infection - leaf necrosis and/or wilting, crown discoloration and severe root necrosis; saprotrophic- dead plants and severely necrotic crowns and roots.

The area under the disease progress curve (AUDPC) was calculated using agricolae version 1.3-7 (de Mendiburu, 2023) in R using disease scores of replicates collected from four timepoints (1, 5, 12, and 21 dpi). Composite statistical tests of AUDPC values of ten replicates per temperature treatment from the two trials were done using the statistical tests used for disease scores.

### 2.2 Root collection

Roots were collected at 1, 5, 12 and 21 dpi from five plants per treatment combination for each experiment. Roots were carefully removed from the pot, rinsed with sterile water to remove the sand, and then blotted dry with a sterile paper towel. Root tissues were divided for RNA extraction and microscopy. For RNA extraction, root tissues were weighed, flash frozen in liquid nitrogen, and stored at −80°C until ready for RNA extraction. For microscopy, fresh root tissues from three of the replicates were stored in glass vials containing 75% ethanol at 10°C.

### 2.3 Microscopy and microsclerotia quantification

Root clearing was done following a modified protocol described by Chowdhury et al. (2014). Root tissues stored in 75% ethanol were patted dry and rinsed with deionized (DI) water. The roots were soaked in 2 ml of 3% KOH for 9 days at 25°C. The KOH solution was removed, and roots were then rinsed with DI water three times. Roots were soaked in 10% H_2_O_2_ for 15 min. The tissues were washed with 5% HCl for 5 min. After decanting the HCl, the roots were stained with lactophenol blue for 5 min and de-stained in acidic glycerol for 15 min. Stained roots were mounted on a glass slide with lactoglycerol for microscopic examination of melanized microsclerotia inside root tissues using Olympus BX53 (Olympus Corporations of the Americas, Central Valley, PA, USA). The Olympus cellSens Standard 4.1 software (Evident Scientific, Inc., Waltham, MA, USA) was used to capture microscopic images and measure root area (mm^2^). Melanized microsclerotia inside root tissues were manually counted. Microsclerotia density was calculated based on the number of internal microsclerotia over the measured root area (mm^2^) and statistical differences in microsclerotia density between different temperatures per timepoint (five to six replicates from two trials) were analyzed using Wilcoxon Rank Sum two-sided test in R with *p* < 0.05. For determining correlations between different disease stages, the Spearman test (*p* < 0.05) was performed in R.

### 2.4 RNA extraction and sequencing

RNA from root samples was extracted following the CTAB-LiCl precipitation protocol described by Yu et al. (2012) with some modifications to adjust for larger amounts of root tissues, and an additional cycle of alcohol rinsing after nucleic acid precipitation. Precipitation in 4 M LiCl at −20°C was performed overnight, and RNA pellets were gently washed with 75% ethanol three times. RNA was then eluted with UltraPure DNase/Rnase-Free Distilled Water (Thermo Fisher Scientific, Waltham, MA, USA). DNAse treatment was done using TURBO DNA-free Kit (Thermo Fisher Scientific, Waltham, MA, USA). Zymo RNA Clean and Concentrator kit (Zymo Research, Irvine, CA) was used to further concentrate and purify RNA samples following the manufacturer’s protocol. The quality of RNA samples was checked on a 1% bleach agarose gel (Aranda et al., 2012), and RNA was quantified using NanoDrop and the Qubit RNA Broad Range Assay (Thermo Fisher Scientific, Waltham, MA). The same methods were employed for extracting and purifying RNA from five *in vitro M. phaseolina* isolate F005 samples that were pooled from five flasks of 3-day-old inoculum for each trial as described above.

After an RNA quality check, three or four replicates of each treatment combination per timepoint for each trial were sent for sequencing at Michigan State University RTSF Genomics Core, MI, USA. cDNA libraries were prepared using the Lexogen QuantSeq 3’ mRNA-Seq Library Prep Kit FWD with the Unique Molecular Identifiers (UMI) Module following manufacturer’s instructions. Completed libraries were checked for quality and quantified using a combination of the Qubit dsDNA HS assay (Thermo Fisher Scientific, Waltham, MA) and Agilent 4200 TapeStation HS DNA1000 assays (Agilent Technologies, Inc., Santa Clara, CA). The libraries were pooled in equimolar quantities for multiplexed sequencing and were quantified using the Invitrogen Collibri Quantification qPCR kit (Thermo Fisher Scientific, Waltham, MA). The pool was loaded onto four lanes of a NovaSeq S4 flow cell. Sequencing was performed in a 1 × 150 bp single read format using a NovaSeq 6000 v1.5 200 cycle reagent kit. Base calling was done by Illumina Real Time Analysis (RTA) v3.4.4 and output of RTA was demultiplexed and converted to FastQ format with Illumina Bcl2fastq v2.20.0.

### 2.5 RNA sequencing reads processing

Raw read quality was checked using FastQC version 0.11.9 (Andrews, 2010). Raw reads were processed by removing UMIs at the 5’ end using UMI-tools version 1.1.2 (Smith et al., 2017), and used cutadapt version 4.0 (Martin, 2011) to remove PolyA tails and 3’ library adapter sequences. Trimmed reads were cleaned using HTStream version 1.3.3^1^. Dual reference mapping was done by first concatenating *M. phaseolina* MP11-12 reference genome (Burkhardt et al., 2019) with the *Fragaria x ananassa* cv. “UCD Royal Royce” reference genome (Hardigan et al., 2021)^2^. Genomes were indexed and mapped using STAR version 2.7.10b (Dobin et al., 2013). Mapping of processed reads to concatenated reference genomes was done using these parameters: “–runMode alignReads –runThreadN 8 –genomeDir ConcatRefs –outSAMtype BAM SortedByCoordinate, –outReads Unmapped Fastx –outFileNamePrefix, –sjdbGTFfile, –outFilter Type BySJout,–outFilterMultimapNmax 20, –alignSJoverhangMin 8, –alignSJDBoverhangMin 1, –outFilterMismatchNmax 999, –outFilterMismatchNoverLmax 0.1, –alignIntronMin 20, –align IntronMax 5000, –alignMatesGapMax 6000, –readFilesCommand zcat, –readFilesIn.” Mapped reads were deduplicated using UMI-tools version 1.1.2 (Smith et al., 2017) and samtools version 1.17 (Danecek et al., 2021). Deduplicated reads were quantified using HTSeq version 2.0.2 (Putri et al., 2022) and the python script used by Jenner and Henry (2022) ^3^ for custom counting. Data quality and mapping statistics were consolidated from this workflow (Supplementary Table 1).

### 2.6 Functional annotation

Specific gene predictions were further performed for *M. phaseolina* in addition to the gene annotations of *M. phaseolina* MP11-12 reference genome (Burkhardt et al., 2019). To predict effectors, SignalP version 6 (Teufel et al., 2022) was used to identify proteins with signal peptides. Proteins with signal peptides were then used to predict effector proteins using EffectorP version 3 (Sperschneider and Dodds, 2022). For predicting carbohydrate-active enzymes (CAZymes), dbCAN version 3 (Zheng et al., 2023) was used. Genes were only determined to be CAZymes if predicted by at least two methods (Zhang et al., 2018). Genes encoding secondary metabolites were identified using antiSMASH 7.0 (Blin et al., 2023).

For the strawberry transcriptome, the same method described above was used to identify CAZymes. For identifying phytohormone-associated genes, phytohormone genes in the Camarosa variety identified previously by Jenner and Henry (2022) were used as references. BLASTn searches were performed against the known phytohormone genes using sequences of differentially expressed genes, and best hits ( > 60% coverage; ≥ 80% identity). GO terms and *Arabidopsis* homologs associated with *Fragaria x ananassa* cv. “UCD Royal Royce” reference genome (Hardigan et al., 2021) were used to determine function of genes. In addition, 17 GO terms related to phytohormone biosynthesis, transport, reception, signaling and regulation were used to identify genes related to phytohormone genes. To identify PR genes, DEGs with Royal Royce gene IDs annotated as PR genes (Vachev et al., 2025) were classified as the corresponding PR gene family. We extracted gene IDs from previous studies on *A. thaliana* (Kim et al., 2022) and *Brassica napus* (Noel et al., 2024) to cross-reference temperature-regulated and immunity-related genes that are homologous to the temperature-regulated strawberry genes we identified in our study.

### 2.7 Gene expression analysis

Custom read counts were filtered using “filterByExpr” function and normalized scaling factors were generated using “calcNormFactors” function with the default Trimmed Mean of M-values (TMM) in edgeR version 4.6.3 (Robinson et al., 2010). The limma package version 3.64.3 (Ritchie et al., 2015) in R was used to perform the subsequent analyses to handle unequal sample sizes per group. The “model.matrix” function was used to generate a design matrix. The design matrix and TMM normalization factors were used in the “voom” function to generate a precision weight for each observation, and normalize and transform counts (log-CPM) (Law et al., 2014, 2016).

Transcriptional changes of *M. phaseolina* and strawberry affected by different variables such as temperatures, timepoints, trials, and disease stages were visualized through principal component analysis (PCA) in R. The “betadisper” function (Anderson, 2006) was performed to test for multivariate homogeneity of group dispersions (variances) in vegan version 2.7-2 (Oksanen et al., 2025). To determine significance of different variables in the transcriptome of *M. phaseolina* and strawberry, a robust test to handle heteroscedastic data and unbalanced replicates was performed using Welch MANOVA-based W*_d_ test (Hamidi et al., 2019) using WdStar 2.4.0 version in R (Alekseyenko and Chen, 2019).

Composite differential gene expression analysis of the two trials was done using limma version 3.64.3 (Ritchie et al., 2015) and contrasts were done between different disease stages, disease stage × temperature combinations and *in vitro* or uninoculated control. Biological replicates per contrast group varied for disease stage groups (at least 8 replicates/contrast group) and disease stage × temperature groups (at least 4 replicates/contrast group). Normalized counts and design matrix were used to fit gene-wise linear models using “lmFit” function. The output object from “lmFit” was used in “eBayes” function to perform variance moderation and add empirical Bayes statistics. The “decideTests” function was used to classify differentially expressed genes. Genes with Benjamini–Hochberg adjusted *p* ≤ 0.05 and | log2 fold change| ≥ 1.5 were considered differentially expressed. Classification of disease stage-specific genes was done by calculating the average z-score of samples categorized to specific disease stages in every DEG. A specific disease stage was algorithmically assigned to a DEG based on the highest average z-score corresponding to a disease stage. To decide whether a DEG is assigned to one or more disease stages, a cut-off of ≥ 0.5 difference in average z-score was used as it differentiates DEGs that were highly upregulated at a single disease stage from the rest of the disease stages. Heatmaps were created using pheatmap version 1.0.13 in R (Kolde, 2025) to visualize gene expression patterns of composite analysis of the two trials. Hierarchically clustered DEGs in heatmaps were based on Spearman correlation distance using factoextra version 1.0.7 (Kassambara and Mundt, 2020). Gene enrichment of specific gene classes in different disease stages was determined through one-sided Fisher’s exact test in R with *p* < 0.05 (Mehta and Patel, 1983; Agresti, 2002).

## 3 Results

### 3.1 More severe necrosis and faster disease progress were associated with high temperatures

Disease severity of *M. phaseolina* infection in strawberry is often evaluated using a 1–5 ordinal scale based on foliar symptoms (Koike et al., 2016; Knapp et al., 2024). This system enables rapid phenotyping of hundreds or thousands of plants for large experiments. As our experiments were smaller, it was feasible to characterize necrosis at the root and crown tissues, which are the primary infection courts of *M. phaseolina*. Using both below- and above-ground symptoms, we classified samples into distinct disease stages: early infection - no aboveground symptoms and no crown or root necrosis; mid-infection - mild stunting, chlorosis or reddening of leaves, root necrosis and/or mild crown discoloration; late infection - leaf necrosis and/or wilting, crown discoloration and severe root necrosis; saprotrophic- dead plants and severely necrotic crowns and roots (Figure 1A and Supplementary Table 2).

Significant differences in disease severity were observed between high and low temperature treatments at 12 days post inoculation (dpi) and 21 dpi (Wilcoxon Rank Sum two-sided test *P* < 0.001) (Figure 1B and Supplementary Figure 1A). At these timepoints, inoculated plants grown under the high temperature treatment had more severe root and crown necrosis, and accelerated plant death. Furthermore, AUDPC values were significantly greater at high temperature (Wilcoxon Rank Sum two-sided test *P* < 0.001), indicating that a more rapid onset of disease occurred in this treatment (Figure 1C and Supplementary Table 3).

To evaluate the associations between temperature and disease stage on production of fungal propagules, we quantified microsclerotia density in root tissues (Supplementary Figure 1B). We only observed significant differences in microsclerotia density between plants incubated at high and low temperature at 21 dpi (Wilcoxon Rank Sum two-sided test *p* = 0.04) (Figure 1D and Supplementary Table 2). Microsclerotia density was significantly correlated with disease stage (Figure 1E) (Spearman *p* < 0.001, ρ = 0.83) but not disease score (Spearman *p* = 0.31, ρ = 0.15) or temperature (Spearman *p* = 0.09, ρ = 0.26).

### 3.2 Differences in *M. phaseolina* and strawberry transcriptomes were greatly influenced by disease stage

**FIGURE 2:**
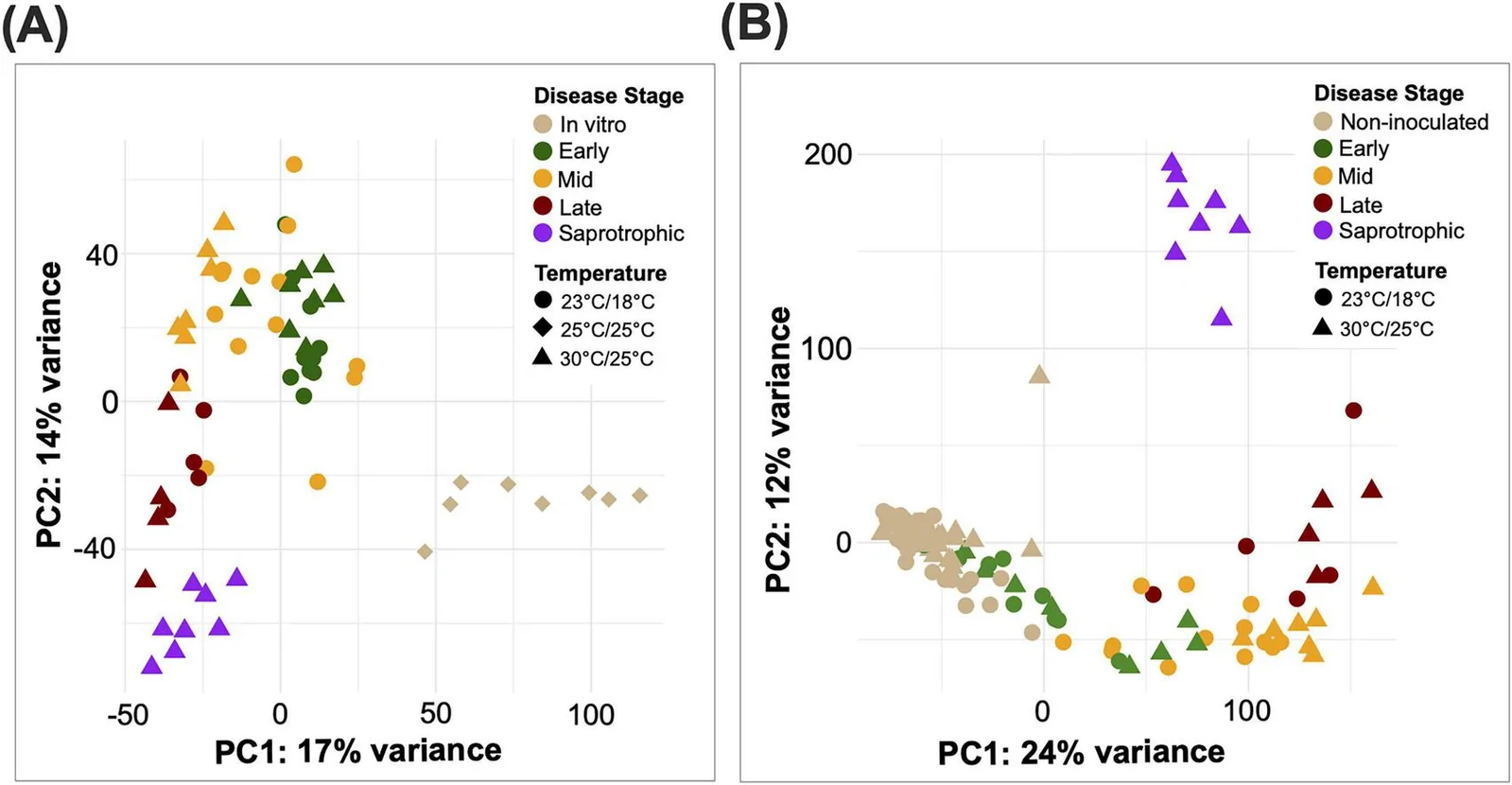
Principal component analysis (PCA) of gene expression in *Macrophomina phaseolina* and strawberry during infection based on composite analysis of two trials. Principal component analysis plot of transcriptional changes influenced by temperature and disease stage in panel **(A)** *Macrophomina*, and **(B)** strawberry. Samples color-coded by disease stage. Different shapes represent different temperatures.

RNAseq and transcriptomic analysis were conducted on samples from two independent experiments. Two susceptible cultivars were used: Monterey in the first trial and San Andreas in the second. Transcriptome data was heteroscedastic in most variables tested (betadisper *p* ≤ 0.05). To determine the effects of variables influencing transcriptional changes in *M. phaseolina* and strawberry despite heteroscedasticity, we conducted a W*_d_ test (Hamidi et al., 2019). The *M. phaseolina* transcriptome was not significantly affected by the strawberry genotype or trial (W*_d_ test FDR-adjusted *p* = 0.11, ω^2^ = 0.01), so the results were pooled and analyzed across two experiments. Disease stage (W*_d_ test FDR-adjusted *p* = 0.002, ω^2^ = 0.49) accounted for the greatest variance explained and samples sharing the same disease stage clustered together in the PCA (Figure 2A). Temperature significantly affected transcriptional changes in *M. phaseolina* (W*_d_ test FDR-adjusted *p* = 0.002, ω^2^ = 0.37).

Similar trends were also seen in the strawberry transcriptome. Disease stage accounted for the greatest variance explained (W*_d_ test FDR-adjusted *p* = 0.002, ω^2^ = 0.39) in the strawberry transcriptome and samples within the same disease stage clustered together (Figure 2B). Temperature (W*_d_ test FDR-adjusted *p* = 0.009, ω^2^ = 0.02) and strawberry genotype (W*_d_ test FDR-adjusted *p* = 0.002, ω^2^ = 0.07) had a significant but small effect on the strawberry transcriptome.

### 3.3 Differentially expressed genes in *M. phaseolina* showed disease stage-specific enrichment

There were 3,539 differentially expressed genes (DEGs) based on pairwise contrasts between disease stages. We classified these genes as being specifically upregulated during one or two disease stages if they had a ≥ 0.5 z-score difference between stages. There were 1,778 DEGs that passed this classification with an unambiguous association with a specific disease stage. To focus our examination on genes that are likely involved in pathogenesis, we only included genes that were upregulated *in planta* compared to *in vitro*, assigned to one or more sequential disease stages and genes that encode for either CAZymes, effectors, or secondary metabolites. In total, 359 DEGs were selected and further examined for specific patterns of expression at different disease stages (Supplementary Table 4).

**FIGURE 3:**
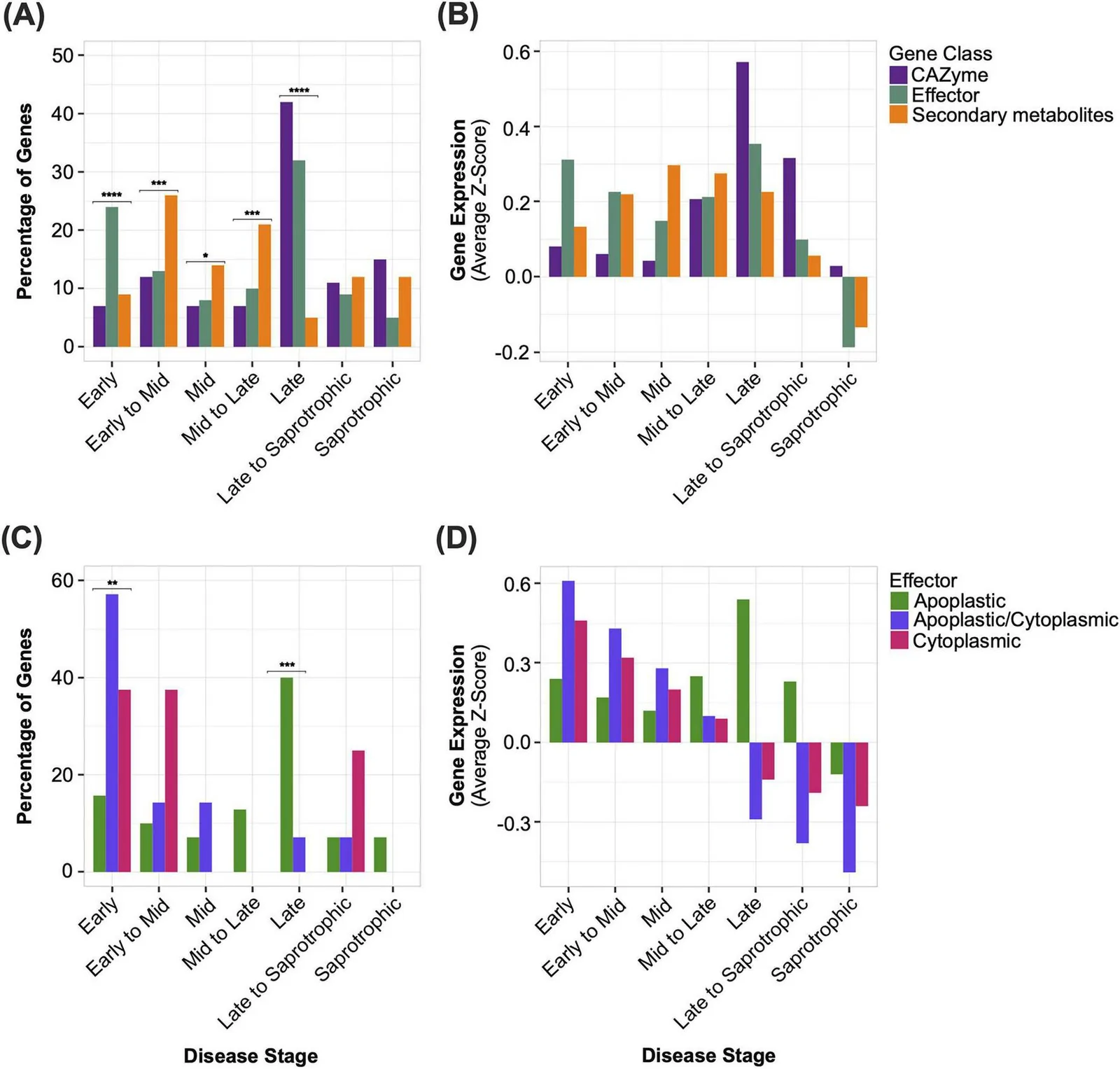
Bar graph of disease stage-specific gene enrichment and expression in *Macrophomina phaseolina* during strawberry pathogenesis based on composite analysis of two trials. **(A)** Percentage of genes depicts the proportion of differentially upregulated genes of a gene class (CAZymes, effectors or secondary metabolite-encoding genes) at each disease stage over the total number of genes in each gene class. **(B)** Gene expression (average z-score) of three gene classes at each disease stage. **(C)** Percentage of genes depicts the proportion of differentially upregulated effector type (apoplastic, apoplastic/cytoplasmic or cytoplasmic effector) at each disease stage over total number of effectors in each type. **(D)** Gene expression (average z-score) of effector-encoding genes at each disease stage. Gene classes share the same color-coding for A and B. Effector types share the same color-coding for C and D. Significant gene enrichment between gene classes per disease stage for A and C based on Fisher’s exact test was annotated with asterisk (*, **, ***, **** indicate *p* < 0.05, 0.01, 0.001, 0.0001 respectively).

At the early infection stage, genes that encode for effectors were significantly enriched (Fisher’s exact test *p* < 0.01) and were highly upregulated compared to other gene classes (Figures 3A,B). These included effectors with a predicted dual apoplastic/cytoplasmic localization (Figures 3C,D). Eleven apoplastic effectors were also upregulated during early infection, some of which were LysM domain and CAP (cysteine-rich secretory proteins, antigen 5, and pathogenesis-related 1 proteins) domain-containing effectors (Supplementary Table 4).

**FIGURE 4:**
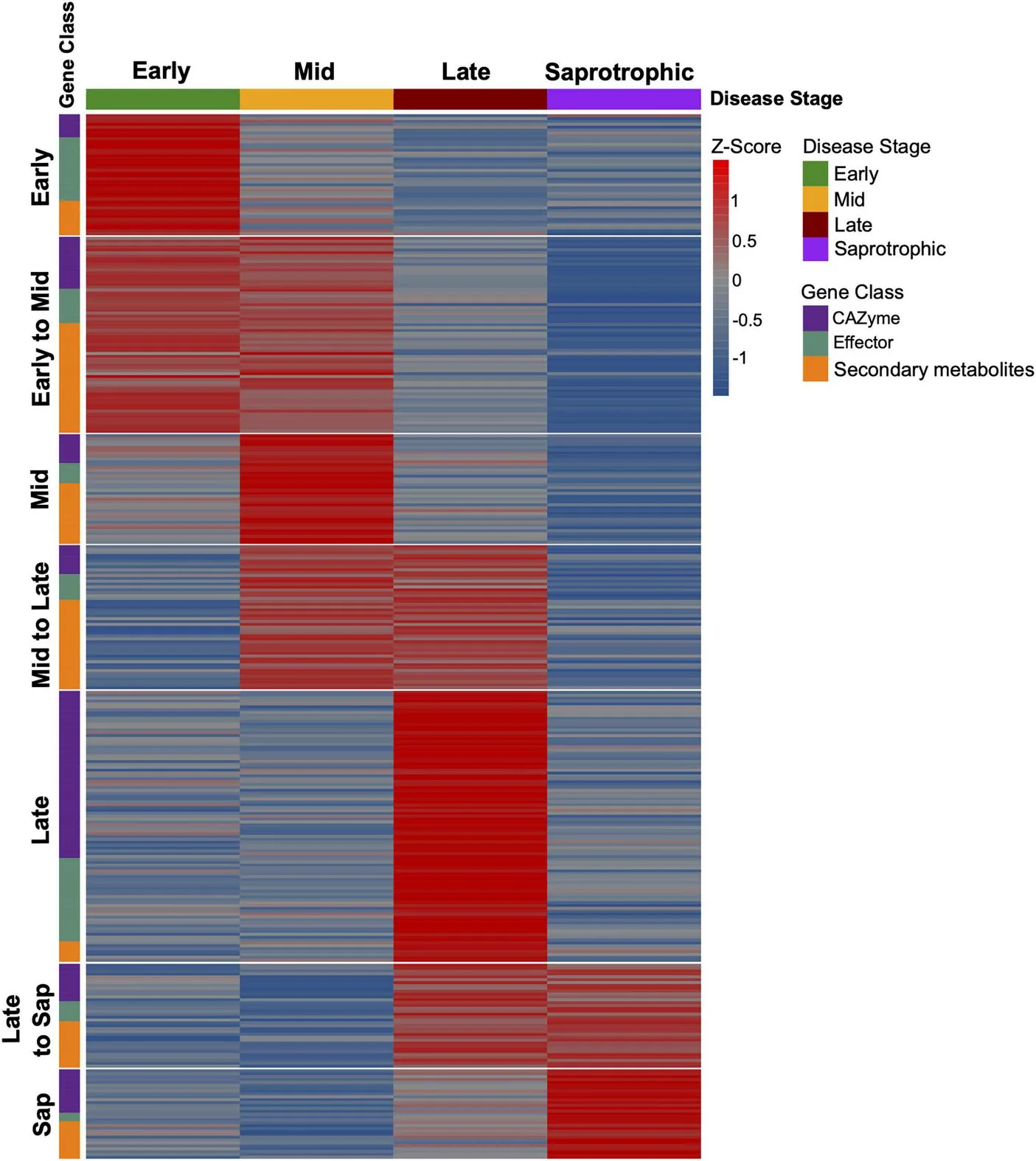
Heatmap of disease stage-specific expression of CAZyme-, effector- and secondary metabolite-encoding genes in *M. phaseolina* based on composite analysis of two trials. Rows depict top 359 disease stage-specific DEGs while columns depict average z-scores of samples classified by disease stage. Gene stage is the assigned disease stage to a DEG based on the highest average z-score corresponding to a disease stage.

The early-to-mid, mid, and mid-to-late infection stages were characterized by significantly enriched expression of secondary metabolite biosynthetic genes (Fisher’s exact test *p* ≤ 0.02; Figures 3A, 4). While the total number of differentially expressed CAZymes did not increase between the mid- and mid-to-late infection stages (Figure 3A), the relative expression (measured by Z-score) increased for these genes (Figure 3B). It is interesting to note that apoplastic effectors were the only class of effectors that were differentially upregulated at mid-to-late infection (Figures 3C,D), which were mostly necrotrophy-associated effectors.

The late infection stage was characterized by the greatest upregulation and enrichment of genes encoding for CAZymes (Fisher’s exact test *p* < 0.01; Figures 3A,B, 4). In terms of effectors, a peak of enrichment and upregulation for apoplastic effectors (Figures 3C,D) was also observed at late infection, most of which were also classified as CAZymes (Supplementary Table 4). The percentage of genes and average level of secondary metabolite gene expression declined at this late infection stage (Figures 3A,B).

The saprotrophic (*in planta*) stage occurred when *M. phaseolina* was feeding on dead host tissues. At this stage, many CAZymes and some secondary metabolite genes were still expressed but declined in level of expression (Figures 3B, 4).

### 3.4 Distinct disease stage-specific enrichment was not observed for phytohormone, CAZyme, or PR genes in strawberry

We identified 13,857 genes that were differentially expressed between different disease stages in strawberry. Again, we focused on genes with known roles in plant-pathogen interactions and found that 763 were classified as plant CAZyme-encoding genes, 157 were genes involved in phytohormone signaling and biosynthesis, and 278 were PR genes. Since 15 of the 16 programmed cell death-associated genes were also associated with phytohormones, we considered these genes as phytohormone-related. Succeeding analyses focused on catalytic CAZymes, phytohormones, and PR genes. After assigning upregulated genes to a specific disease stage, 427 genes had an unambiguous disease stage classification and were also classified as either plant CAZyme, phytohormone or PR genes (Supplementary Table 5).

There was no distinct disease stage-specific enrichment among plant CAZyme, phytohormone and PR genes in strawberry (Supplementary Figure 2A). However, all three plant gene classes had the highest percentage of genes upregulated at mid-to-late infection compared to other disease stages. It is notable that the average induction of PR and phytohormone-related genes have distinct patterns at different disease stages (Supplementary Figures 2B, 3). Furthermore, examining the enrichment of different phytohormone subcategories revealed ethylene (ET)-related genes were highly enriched at early infection stage (Fisher’s exact test *p* < 0.01) while jasmonic acid (JA)-related genes were enriched at mid- to late infection (Fisher’s exact test *p* < 0.01) (Supplementary Figure 2C). During necrotrophic phase at mid-infection until late infection, JA-related genes had the highest gene expression among the four phytohormones (Supplementary Figure 2D).

### 3.5 High temperature upregulated and accelerated expression of pathogenicity-associated genes in *M. phaseolina* while suppressing plant defense-related genes

**FIGURE 5:**
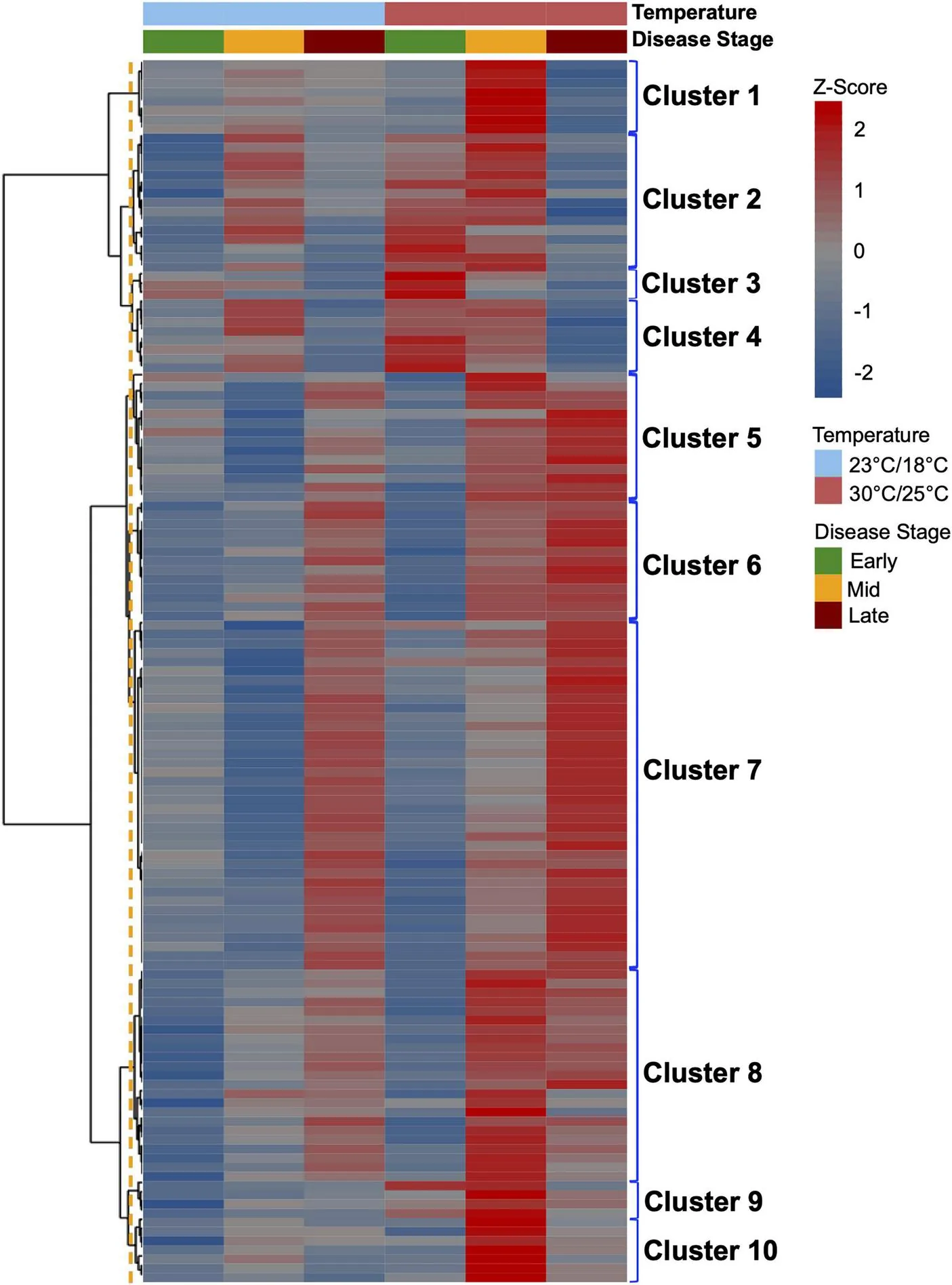
Heatmap of differentially expressed genes of *M. phaseolina* at high temperature (30°C) at different stages of infection based on composite analysis of two trials. Rows depict 133 disease stage × temperature DEGs while columns depict average z-scores of samples classified by disease stage. DEGs were hierarchically clustered based on Spearman correlation distance. Dashed yellow line depicts the cut-off for determining the 8 clusters in the dendrogram marked in blue brackets.

We further examined the effects of temperature in the transcriptome of *M. phaseolina* during root infection through differential expression analysis between temperatures at each disease stage. Since disease progress was accelerated at high temperature, we focused on *M. phaseolina* genes that were highly upregulated at 30°C relative to 23°C to identify genes that may be involved in the accelerated disease progress at high temperature. Among the 3,516 disease stage × temperature DEGs, 133 genes were upregulated under high temperature at specific disease stages (Figure 5). Most of these genes (over 77%) had greater induction at high temperature in the mid-infection disease stage (Figure 5).

To better understand the temperature-influenced pattern of gene expression, we hierarchically clustered these 133 genes. This produced ten clusters, and six clusters were comprised of disease stage-specific genes that were mostly upregulated at 30°C compared to the 23°C within the same disease stage (clusters 1,3, 4, 8, 9, and 10). Meanwhile, four clusters (clusters 2,5,6,7) comprising most of the high temperature-associated genes were upregulated at an earlier stage in the high temperature treatment (Figure 5). For instance, cluster 2 DEGs were already induced at the early stage of infection under 30°C when the peak upregulation of these genes was observed at mid-infection stage at 23°C (Supplementary Table 6). A similar trend was observed in DEGs found in clusters 5, 6 and 7 where significant upregulation of genes was observed at 30°C during mid-infection when these DEGs were mostly late stage-specific DEGs.

We also examined DEGs in the strawberry transcriptome that, within a disease stage, had higher expression at 23°C than at 30°C, and vice versa. The first category represented putative immunity-related genes suppressed by high temperature, while the second category comprised possible susceptibility genes. We identified 222 temperature-regulated DEGs in strawberry during different infection stages of *Macrophomina* infection (Supplementary Figure 4). Most of these temperature-regulated genes fell into the first category: putative immunity-related genes suppressed by high temperature. Of these genes, 132 genes were less repressed at 23°C (less negative z-score) while 48 genes were more induced at 23°C than at 30°C (Supplementary Table 7). Meanwhile, 42 genes highly upregulated at 30°C were mostly plant stress response genes and possible susceptibility genes.

## 4 Discussion

Our findings support the hypothesis that *M. phaseolina* has a hemibiotrophic lifestyle during strawberry infection. Based on visual disease symptoms and transcriptional changes at different stages of infection, we distinguished two phases of *M. phaseolina* infection based on the presence of disease symptoms: a biotrophic phase (early infection) when plants were asymptomatic, and a necrotrophic phase (mid and late infection) when plants exhibited wilting, root or crown necrosis, and death. Our differential gene expression analysis for each disease stage led to the identification of thousands of DEGs across different disease stages, which we categorized based on one or two disease stages in which they showed highest expression. We focused on three categories of fungal genes previously shown to characterize biotrophic vs. necrotrophic stages of fungus-plant interactions: effectors, secondary metabolite biosynthesis, and CAZymes.

We observed a biotrophic phase during the early stage of *M. phaseolina* infection when no symptoms were observed in strawberry plants, which mostly consisted of samples collected at 1 dpi. Several studies have documented a biotrophic phase in hemibiotrophic plant pathogenic fungi within 24 h post inoculation (Kabbage et al., 2015; Chowdhury et al., 2017). That the biotrophic phase lasted only 1 to 5 days under growth chamber conditions, compared to the long asymptomatic phase that Pedroncelli et al. (2025), could be due to the more conducive conditions for infection along with high inoculum levels that we have used in our study. At the early infection stage, when no symptoms were observed in strawberry plants, genes encoding for effectors were most enriched and highly expressed in the *M. phaseolina* transcriptome, as has been shown for the biotrophic stage of *Colletotrichum* spp. (O’Connell et al., 2012). Specifically, we identified LysM domain- and CAP domain-containing genes among apoplastic effectors that were highly upregulated during the early infection stage of *M. phaseolina*, as was similarly reported in *S. sclerotiorum*, *C. higginsianum*, and *Puccinia striiformis* f.sp. *tritici* (Takahara et al., 2016; Chittem et al., 2020; Zhao et al., 2023). The upregulation of these apoplastic effectors in our study previously reported to be associated with biotrophy supports our characterization of early infection as the biotrophic phase of *M. phaseolina* in strawberry.

The necrotrophic phase of *M. phaseolina* encompassed mid- and late-infection stages when root and/or crown necrosis was observed. RNAseq is conducted on a population of cells, some of which may be still in the early stages of necrotrophy and others at late stages, and the infection process is asynchronous, with plants exhibiting faster or slower disease progression due to random, uncontrolled variance. These factors make it impossible to define the exact timepoint corresponding to a “switch” to necrotrophy. However, the mid-infection stage was both phenotypically and transcriptomically distinct from the biotrophic phase, based on increased microsclerotia density, visible necrosis, and enrichment of DEGs encoding for biosynthesis of secondary metabolites, and down-regulation of effector gene expression. Many secondary metabolites act as toxins and advance necrotrophic growth (Horbach et al., 2011; Stergiopoulos et al., 2013; Choquer et al., 2005), and we hypothesize that their increased expression at the mid-infection stage indicates the switch to necrotrophy.

At mid-to-late and late disease stages, fungal necrotrophy-associated effectors were up-regulated, including necrosis-inducing protein and cerato-platanin, which have been associated with necrotrophy in *S. sclerotiorum* (Yang et al., 2018; Chittem et al., 2020). At the late infection stage, 86% of induced effectors were also classified as fungal CAZymes, which aligned with the peak of CAZyme enrichment and expression at this stage. The enrichment of fungal CAZymes at the late stage was mostly comprised of glycoside hydrolases (GHs) and is similar to reports that CAZymes are enriched during late infection by hemibiotrophic pathogens like *C. higginsianum* and *C. graminicola* (O’Connell et al., 2012). Fungal glycoside hydrolases break down carbohydrates and polysaccharides promoting plant tissue necrosis and accelerated fungal growth (Rafiei et al., 2021). Glycoside hydrolase families such as GH7, GH10, GH11, and GH12 are known to act as effectors in hemibiotrophic fungi and oomycetes (Nguyen et al., 2011; Van Vu et al., 2012; Gui et al., 2017; Tan et al., 2020; Liu L. et al., 2021). Of GHs previously documented to be fungal effectors, we observed DEGs belonging to GH7, GH10 and GH12 upregulated at late infection in our study. Our transcriptome analysis suggests that *M. phaseolina* uses necrotrophy-associated effectors and fungal CAZymes to maximize necrotrophic pathogenesis at the late infection stage.

We also profiled the molecular events of susceptible strawberry interactions with *M. phaseolina*. Compared with the fungal transcriptome, the strawberry transcriptome did not have as pronounced disease-stage specific enrichment of the specific categories of genes: CAZymes, phytohormone- related and PR genes. However, we observed that the abundant upregulation of plant CAZymes at mid-to-late stage was mostly comprised of plant glycosyltransferases, which are known to play a role in cell wall biosynthesis (Scheible and Pauly, 2004) and plant secondary metabolism that is pivotal in stress tolerance and defense (Vogt and Jones, 2000). Most of the PR genes upregulated at mid-to-late stage were PR9 (secretory peroxidase) genes, known to be important in plant cell strengthening through lignin deposition during pathogen invasion (Kaur et al., 2022). Furthermore, it was notable that despite not having significant enrichment of phytohormones across different disease stages, there was disease stage-specific enrichment within the different classes of phytohormone-related genes in strawberries. We observed enrichment of ethylene signaling genes, including homologs of Ethylene Response Sensor 1 (*ERS1*) (Hall et al., 2000) and Ethylene Response Factor 1 (*ERF1*) (Lorenzo, 2003) during the early infection stage. Ethylene plays a crucial role in modulating plant defense signaling pathways, including those regulated by salicylic acid and jasmonic acid, and may have a dual role in either enhancing disease susceptibility or resistance against pathogens (van Loon et al., 2006; Broekgaarden et al., 2015). Since our transcriptomic study only used susceptible strawberry varieties, the ethylene signaling at the early stage of infection was insufficient for preventing disease caused by *M. phaseolina*. At the mid-infection to late disease stage, JA-related genes were highly enriched, and were induced more strongly than other phytohormone-related genes. JA is associated with resistance to necrotrophic pathogens and insects (Zhang et al., 2017). Though induction of these genes did not result in resistance to *M. phaseolina*, the expression of these genes supports our hypothesis that the necrotrophic phase of the pathogen encompasses mid-infection to late disease stage.

Our study demonstrated that accelerated disease progress and plant mortality was highly associated with high temperature. These findings were similar to previous controlled environment (Fang et al., 2011) and greenhouse studies (Zveibil et al., 2012) on *M. phaseolina* infection of strawberries, where increased disease severity scores and more rapid plant death were observed under high temperatures (30–32°C). Similarly, 5 years of field trials also demonstrated that higher temperatures are associated with greater *M. phaseolina* disease severity in strawberry (Wang Y. C. et al., 2024).

Microsclerotia development has been associated with host cell death and therefore serves as an indicator for necrotrophy (Short et al., 1978; Shirai and Eulgem, 2023). In our study, microsclerotia density was significantly higher at 30°C than the 23°C treatment at 21 dpi and was numerically higher at all other timepoints. There was high variability in microsclerotia density in strawberry roots; variability was also previously noted in soybean (Short et al., 1978). High variability of microsclerotia density in strawberry roots may be attributed to variation of microsclerotia growth in different strawberry root fragments (primary and secondary roots) as well as the position from which the root fragments were collected along the length of the pooled root tissues. Furthermore, we observed a strong positive association between microsclerotia density and later disease stages when necrosis was already observed. Taken together, high temperature accelerates disease progress, leading to more abundant microsclerotia production in later disease stages.

We have identified temperature-regulated genes in both *M. phaseolina* and strawberry. We hypothesized that high temperature favors *M. phaseolina* infection in strawberry through the upregulation of fungal effectors, fungal pathogenicity-associated genes, plant susceptibility factors and the suppression of plant defense response genes, thereby resulting in more severe necrosis, accelerated disease progress, and plant death in strawberry. We observed two types of DEGs upregulated at high temperatures in *M. phaseolina*. The first type were those DEGs that were differentially upregulated at 30°C compared to 23°C within the same disease stage. These DEGs included CAZymes and effectors including cerato-platanin and secondary metabolites associated with necrotrophy and pathogenicity (Pazzagli et al., 2014; Yang et al., 2018), as well as oligopeptide, ABC (ATP-Binding Cassette), and Major Facilitator Superfamily (MFS) transporters, and cytochrome P450s that have played a role in fungal virulence and pathogenicity (Chagué et al., 2009; Perlin et al., 2014; O’Mara et al., 2023; Wang Y. et al., 2024). These genes could have contributed to more severe symptoms within the same disease stage or represent the beginning of an early progression to the next disease stage.

The second type of high temperature-upregulated DEGs in *M. phaseolina* were those that were induced at an earlier stage, which encompassed 60% of the DEGs at high temperature. A noteworthy example was the induction of three mid-infection-specific MFS transporters that were already upregulated at 30°C during the early stage. MFS transporters known to play an essential role in virulence (Lin et al., 2018) and protection against plant defense-related compounds during infection (Chen et al., 2021; Liu N. et al., 2021). There were more late infection stage-specific genes that were already upregulated at 30°C during mid-infection, more than half of which were genes encoding CAZymes (mostly GHs), effectors and secondary metabolites. The higher and more rapid induction of these genes at high temperatures could be either a cause or effect of more rapid disease progression: it is possible that a “mid-infection” sample at 30°C represents a more advanced infection within the same mid-infection bin. On the other hand, upregulation of these genes during early infection could pre-empt plant defense response, thereby giving *M. phaseolina* an advantage in advancing infection. The upregulation of these genes working in concert at an earlier stage, is a compelling explanation for the accelerated disease progress under high temperature.

We further tested the second part of our hypothesis that high temperatures could upregulate expression of plant susceptibility factors and suppress plant defense-related genes. Some DEGs upregulated at high temperature were associated with abiotic stress response, such as heat shock proteins (Ul Haq et al., 2019), tetratricopeptide repeat (TPR)-like superfamily proteins (Liu et al., 2024), and F-Box proteins (Li et al., 2018). These may represent pathogen-independent plant stress responses related to high temperature. On the other hand, we also observed that sugar transporters in strawberry were upregulated at 30°C during the late infection stage. Sugar transporters regulate carbon partitioning in plants and were found to be susceptibility factors during bacterial and fungal infection (Garcia-Ruiz et al., 2021; Lei et al., 2025). Aside from sugar transporters, we also observed significant upregulation of an ethylene-forming enzyme at mid-infection stage at 30°C. Ethylene-forming enzyme is involved in the biosynthesis of ethylene, and in turn, ethylene plays a dual role in plant defense response during inoculation and susceptibility to hemibiotrophic fungi when present after infection (Robison et al., 2001; Shu et al., 2024). Taken together, these sugar transporters and ethylene-forming enzyme could be possible susceptibility factors in the *M. phaseolina* – strawberry interaction. Future investigations comparing resistant and susceptible strawberry varieties are needed to determine which or if any of these possible susceptibility factors when upregulated, diminish resistance at elevated temperatures. Disruption of these genes through transformation or gene editing may unravel the mechanisms of susceptibility to *M. phaseolina*.

Most of the temperature-regulated genes identified in strawberry were genes that were highly upregulated at 23°C or downregulated at 30°C. Elevated temperatures have been reported to inhibit resistance gene expression (Wang et al., 2009; Zhu et al., 2010) and disrupt SA-mediated basal immunity (Kim et al., 2022), leading to plant susceptibility. Downregulation of defense-related genes such as PR-2 (β-1,3-endoglucanase), PR-5 (thaumatin-like), chalcone synthase, peroxidase, and lipoxygenase genes was observed under high temperatures in a susceptible chickpea variety during Macrophomina dry root rot infection (Sharath Chandran et al., 2021). In connection with these previous reports, we found leucine-rich repeat protein kinases and homologs of immunity-related genes in *A. thaliana* (Kim et al., 2022) and *Brassica napus* (Noel et al., 2024) that were only upregulated at lower temperatures. We also found PR genes, specifically two PR-10 genes to be less suppressed under low temperature in the susceptible strawberry varieties we used in this study. Pathogenesis-related 10 (PR-10) plays multi-functional role in plant growth and development, as well as plant defense response such as ribonuclease (RNase) activity and phytohormone-mediated defense signaling (Besbes et al., 2019; Kumari et al., 2026). We hypothesize that downregulation of these genes at high temperatures was likely a factor that contributed to susceptibility to *M. phaseolina*.

Future studies that involve transformation to overexpress these putative immunity-related genes in the susceptible strawberry varieties under high temperatures are needed to validate the role of these genes in plant resistance.

## 5 Conclusion

Our study provides a comprehensive examination of the effects of high temperature on disease progress and transcriptional changes during the complete infection cycle of *M. phaseolina*. At high temperatures, disease progress was significantly accelerated. Our results supported the characterization of *M. phaseolina* as a hemibiotroph, due to the lack of necrosis and expression of key biotrophy-related genes during early infection. Meanwhile, the necrotrophic phase of the pathogen corresponded with increased microsclerotia density, root and crown necrosis, and upregulation of fungal genes encoding for secondary metabolite and CAZymes. Transcriptional changes in the susceptible strawberry varieties we used in our study identified molecular events during a compatible interaction with the pathogen. Future investigation comparing resistant and susceptible strawberry varieties, or gene knockout and complementation experiments are needed to confirm susceptibility factors to *M. phaseolina*. Furthermore, our study presented possible mechanisms underlying the more severe symptoms and accelerated disease progress during *Macrophomina* infection in strawberry at elevated temperatures. Under the high temperature treatment, *M. phaseolina* pathogenicity-related genes were induced earlier, putative plant susceptibility factors were upregulated, and strawberry defense-related genes were down-regulated. To our knowledge, our study is the first molecular characterization of the *M. phaseolina-*strawberry interaction, providing insights that can be translated to other crops within the host range of *M. phaseolina*, especially cooler weather crops that are grown in a warming climate. Possible susceptibility genes identified in our study could be targets for mitigating the devastating effects of high temperatures through resistance breeding or biotechnology.

## Data Availability

Raw sequencing reads were deposited in the NCBI SRA database with BioProject accession PRJNA1307604.

## Footnotes

1
https://github.com/s4hts/HTStream

2
https://datadryad.org/stash/dataset/doi:10.25338/B8TP7G

3
https://github.com/pmhenry/Publications/tree/master/Frag_Fof_TAGseq_Jenner_2020/HTSeq-TAG-Counts
