# Supplementary material for: Macrophomina phaseolina expresses sequential waves of effector, secondary metabolite, and CAZyme genes during strawberry infection, suggesting a hemibiotrophic lifestyle
Source: Front Microbiol. 2026 Aug 31;17:1856252. doi: 10.3389/fmicb.2026.1856252 (PMC13572228; doi:10.3389/fmicb.2026.1856252)
Supplement: Supplementary file 1 [file Data_Sheet_1.pdf]

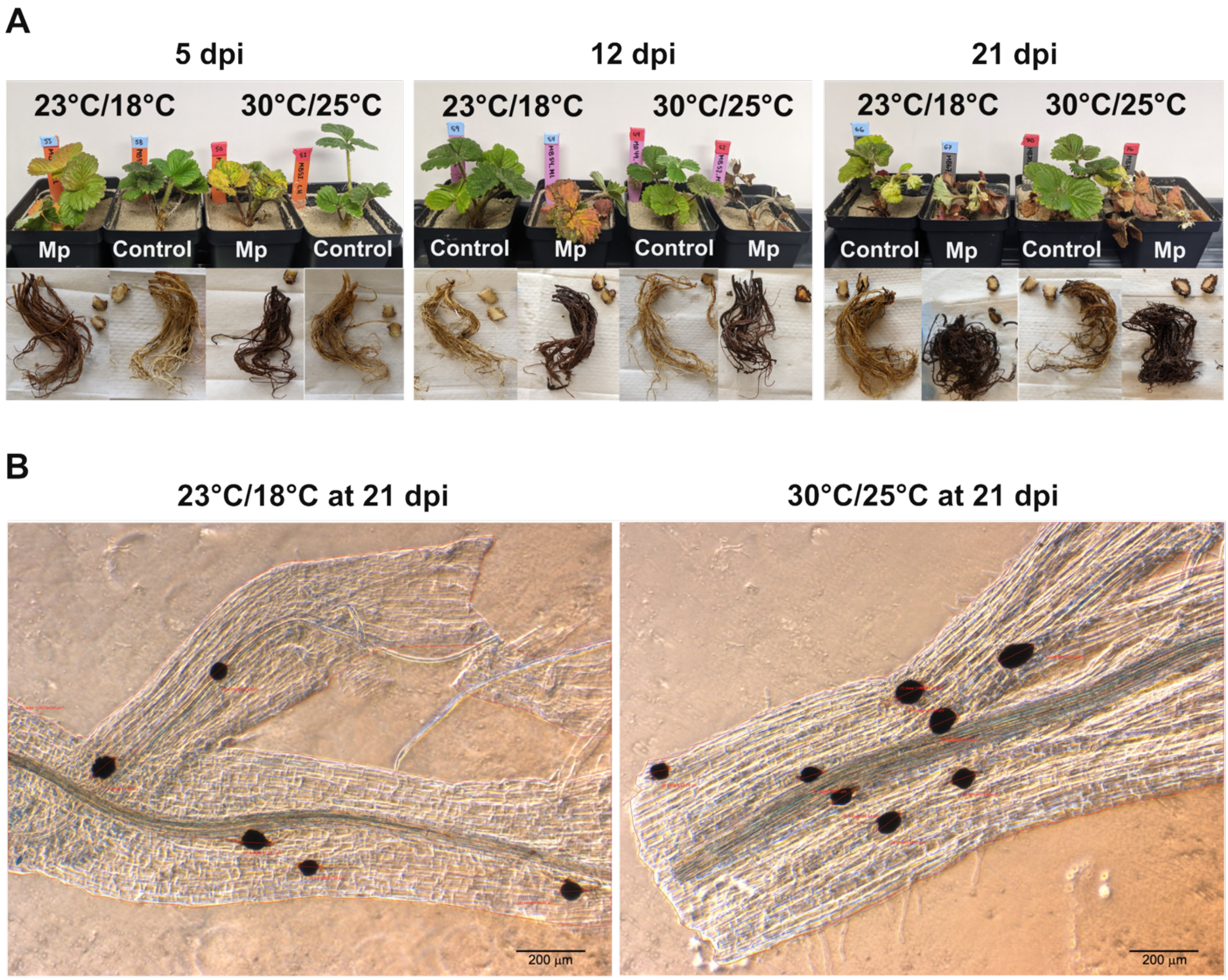

Supplementary Figure S1. Severity of *Macrophomina* crown and root rot disease and microsclerotia growth in strawberry roots. A) Shoot, crown, and root images of infected strawberry plants at different temperatures per timepoint. B) Representative microscopic images of quantified microsclerotia in strawberry roots under high (30°C day/25°C) and low (23°C day/18°C) temperatures at 21 dpi. Images acquired on an Olympus BX53 microscope (4× objective) using Olympus cellSens Standard 4.1 software. Scale bar = 200 µm.
