# Supplementary material for: Macrophomina phaseolina expresses sequential waves of effector, secondary metabolite, and CAZyme genes during strawberry infection, suggesting a hemibiotrophic lifestyle
Source: Front Microbiol. 2026 Aug 31;17:1856252. doi: 10.3389/fmicb.2026.1856252 (PMC13572228; doi:10.3389/fmicb.2026.1856252)
Supplement: Supplementary file 2 [file Data_Sheet_2.pdf]

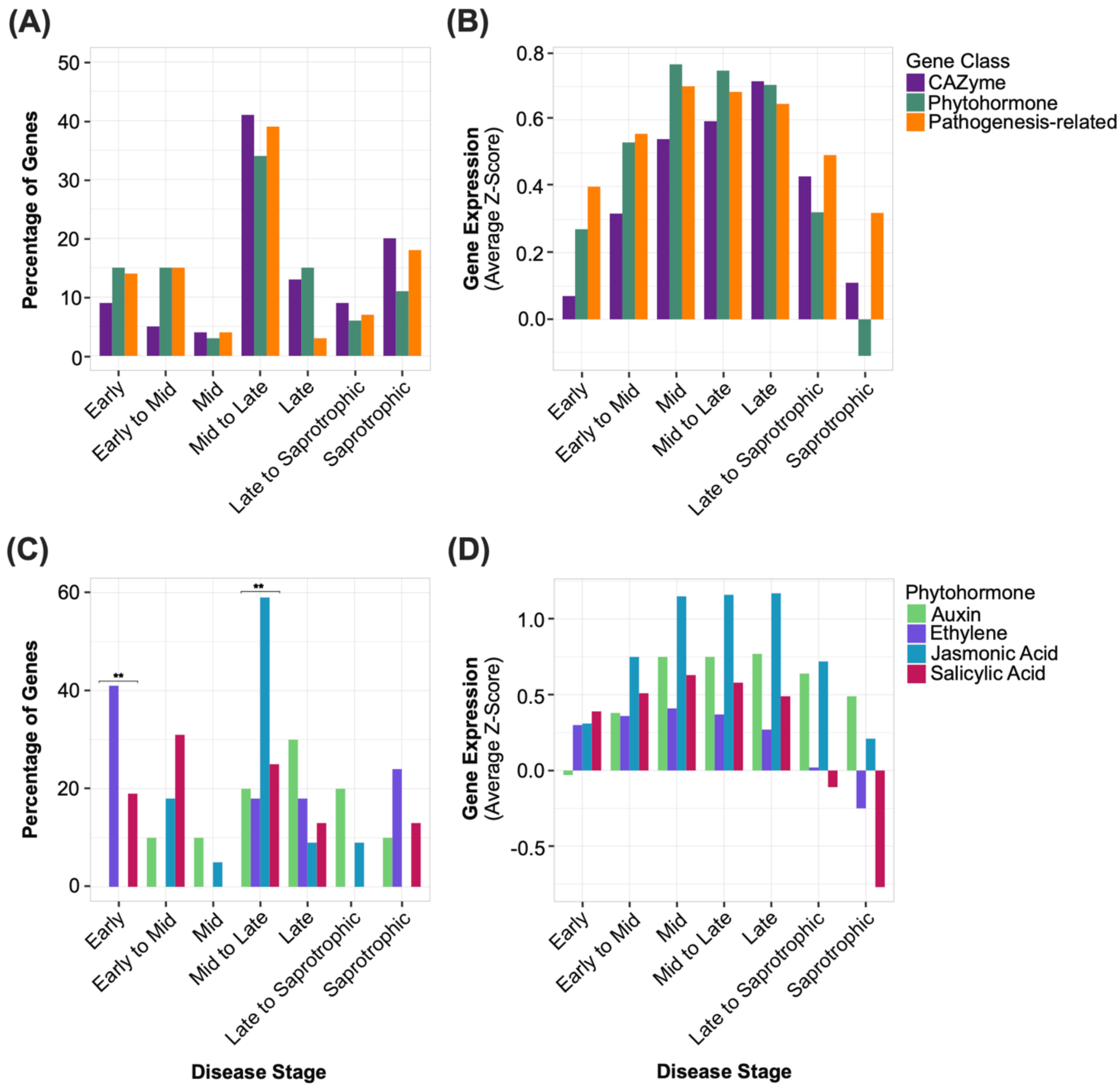

Supplementary Figure S2. Bar graph of disease stage-specific gene enrichment and expression of strawberry genes during a *Macrophomina phaseolina* infection. A) Percentage of genes depicts the proportion of differentially upregulated genes of a gene class (CAZymes, phytohormones or pathogenesis-related genes) at each disease stage over total number of genes in each gene class. B) Gene expression (average z-score) of three gene classes at each disease stage. C) Percentage of genes depicts the proportion of differentially upregulated phytohormone-related genes at each disease stage over the total number of genes in each type. D) gene expression (average z-score) of phytohormone-related genes at each disease stage. Gene classes share the same color-coding for A and B. Phytohormone types share the same color-coding for C and D. Significant gene enrichment between gene classes per disease stage for A and C based on Fisher's exact test was annotated with asterisk (*, **, ***, **** indicate $p <$ 0.05,0.01,0.001,0.0001 respectively).
