# Supplementary material for: Macrophomina phaseolina expresses sequential waves of effector, secondary metabolite, and CAZyme genes during strawberry infection, suggesting a hemibiotrophic lifestyle
Source: Front Microbiol. 2026 Aug 31;17:1856252. doi: 10.3389/fmicb.2026.1856252 (PMC13572228; doi:10.3389/fmicb.2026.1856252)
Supplement: Supplementary file 3 [file Data_Sheet_3.pdf]

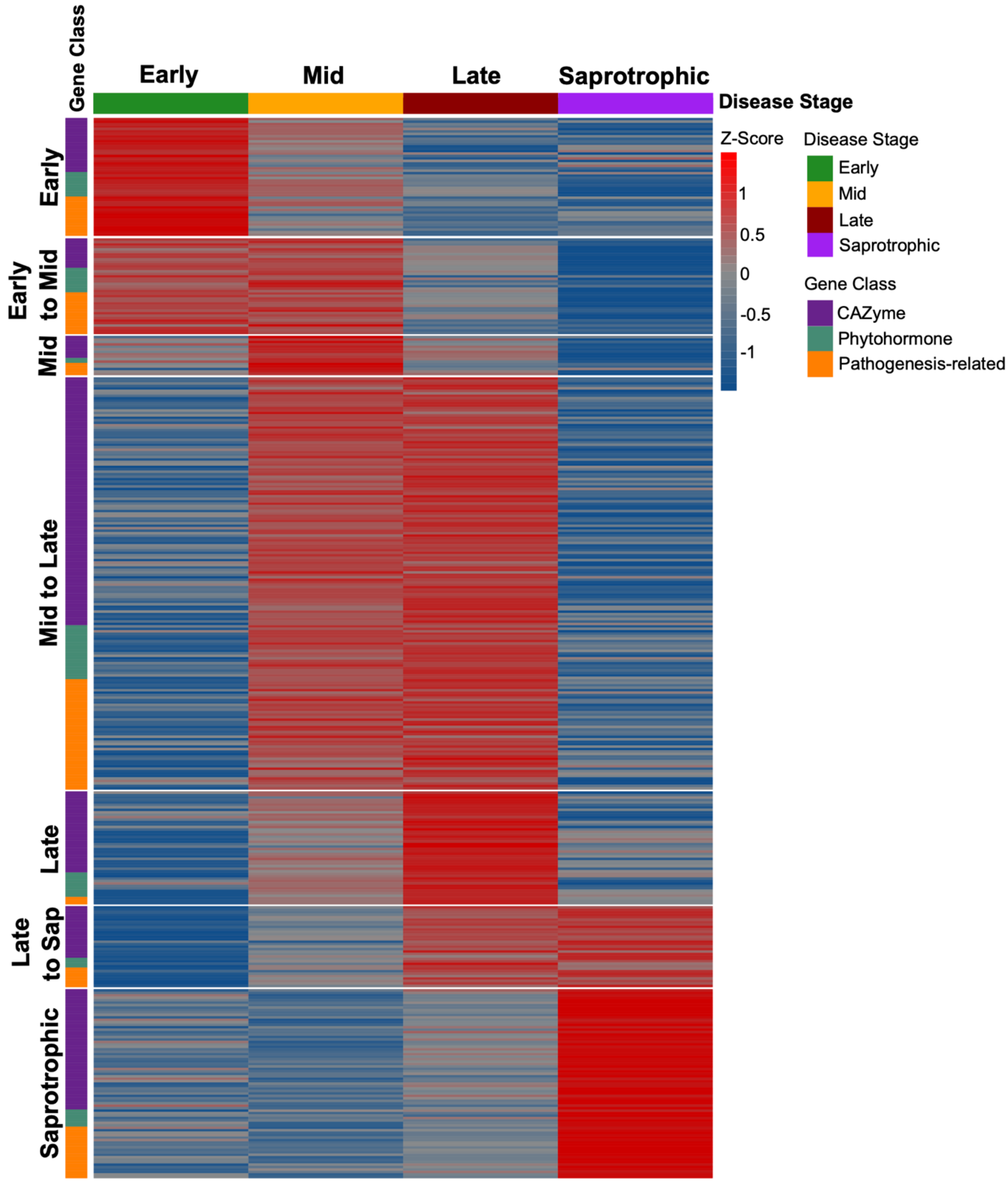

Supplementary Figure S3. Heatmap of disease stage-specific expression of genes encoding CAZymes, phytohormones and pathogenesis-related in strawberry during *M. phaseolina* infection. Rows depict top 427 disease stage-specific DEGs while columns depict average z-scores of samples classified by disease stage. Gene stage is the assigned disease stage to a DEG based on the highest average z-score corresponding to a disease stage.
