# Supplementary material for: Macrophomina phaseolina expresses sequential waves of effector, secondary metabolite, and CAZyme genes during strawberry infection, suggesting a hemibiotrophic lifestyle
Source: Front Microbiol. 2026 Aug 31;17:1856252. doi: 10.3389/fmicb.2026.1856252 (PMC13572228; doi:10.3389/fmicb.2026.1856252)
Supplement: Supplementary file 4 [file Data_Sheet_4.pdf]

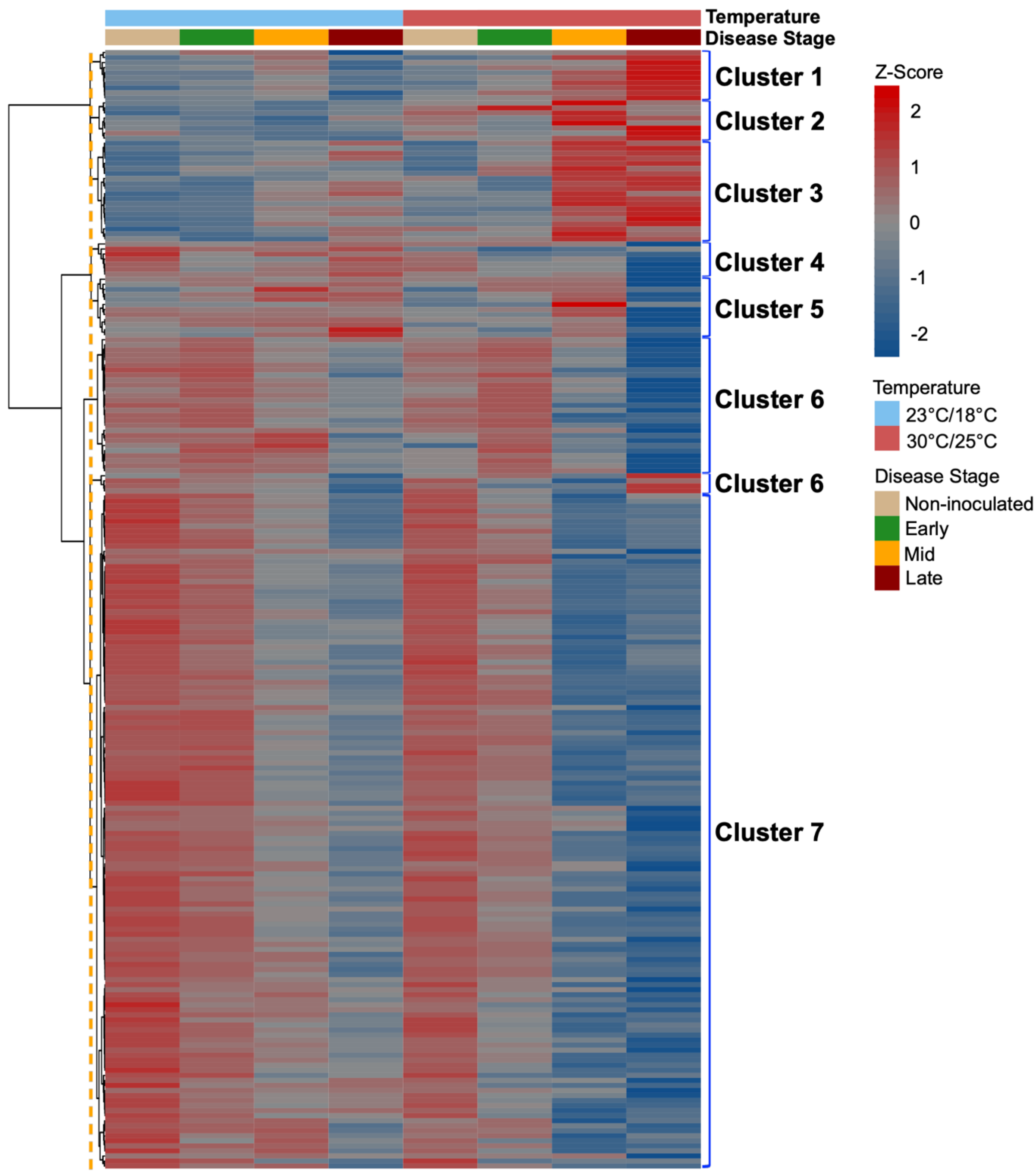

Supplementary Figure S4. Heatmap of differentially expressed genes of strawberry at different temperatures at different stages of infection. Rows depict 222 disease stage x temperature DEGs while columns depict average z-scores of samples classified by disease stage. DEGs were hierarchically clustered based on Spearman correlation-based distance. Dashed yellow line depicts the cut-off for determining the 8 clusters in the dendrogram marked in blue brackets.
